# Development of a neuroimaging biomarker of the pro‐coagulant state in Alzheimer's disease: The BioClotAD Project

**DOI:** 10.1002/alz70856_100174

**Published:** 2025-12-25

**Authors:** Nicolás Lamanna‐Rama, Marta Casquero‐Veiga, Carlos Ceron, Gorka Sobrino, Irene Fernandez‐Nueda, Susanne Kossatz, Dag Sehlin, Manuel Desco, Beatriz Salinas, Marta Cortes‐Canteli

**Affiliations:** ^1^ Consejo Superior de Investigaciones Científicas ‐ Centro Internacional de Neurociencia Cajal (CSIC ‐ CINC), Alcalá de Henares, Madrid, Spain; ^2^ Spanish National Center for Cardiovascular Research (CNIC), Madrid, Madrid, Spain; ^3^ Instituto de Investigación Sanitaria Fundación Jiménez Díaz (IIS‐FJD), Madrid, Madrid, Spain; ^4^ Advanced Imaging Unit, Spanish National Center for Cardiovascular Research (CNIC), Madrid, Madrid, Spain; ^5^ Instituto de Investigación Sanitaria Gregorio Marañón (IiSGM), Madrid, Madrid, Spain; ^6^ German Cancer Consortium ‐ German Cancer Research Center (DKTK ‐ DKFZ), Heidelberg, Baden‐Württemberg Land, Germany; ^7^ Nuclear Medicine, Klinikum rechts der Isar, Technical University Munich (TUM), Munich, Upper Bavaria, Germany; ^8^ Center for Translational Cancer Research (TranslaTUM), Technical University Munich, Munich, Upper Bavaria, Germany; ^9^ Department of Public Health and Caring Sciences, Molecular Geriatrics, Rudbeck Laboratory, Uppsala University (UU), Uppsala, Uppsala, Sweden; ^10^ Department of Bioengineering, Universidad Carlos III de Madrid (UC3M), Leganés, Madrid, Spain; ^11^ Centro de Investigación Biomédica en Red de Salud Mental (CIBERSAM), Madrid, Madrid, Spain

## Abstract

**Background:**

Alzheimer's disease (AD) is the most common form of dementia^[1]^. Despite the advances in the understanding of its main neuropathological hallmarks, a significant gap remains in comprehending this multifactorial neurodegenerative disorder. Contributing factors to AD include chronic vascular dysregulation and a prothrombotic milieu, promoting fibrin accumulation in brain vessels^[2]^. Fibrin, the main protein component of blood clots, is significantly increased in 60% of AD‐patients’ brains^[3]^. Furthermore, its strong interaction with amyloid‐β promotes the production of degradation‐resistant clots^[2]^. This prothrombotic milieu intensifies hypoperfusion, neurodegeneration and blood‐brain barrier (BBB) disruption^[4]^, but not in all patients. Early detection could help identify patients which might benefit from anticoagulant therapies^[5]^. The BioClotAD Project aims to develop an imaging biomarker based on a fibrin binding probe (FBP)^[6]^ to non‐invasively identify AD's pro‐coagulant state.

**Method:**

BioClotAD involves four partners in three European countries with complementary expertise, using extensive *in vitro*, *ex vivo*and *in vivo* assays, including AD animal models and human AD brain samples. Our project is based on three blocks: 1) Testing the FBP to *in vivo* detect the cerebral occlusions by nuclear imaging. 2) Detecting fibrin deposits inside the brain parenchyma with FBP coupled to a transferrin receptor antibody (FBP‐TfR) to facilitate BBB crossing^[7]^. FBP‐TfR will be labelled for optical and nuclear imaging. 3) Validating the most promising FBP probes in frozen brain samples of AD patients by autoradiography or fluorescence microscopy.

**Result:**

BioClotAD provides feasible neuroimaging strategies to *in vivo* detect the fibrin cerebral accumulation of AD models and identify the specific regional distribution in the brain. Our project will set the basis for future clinical trials on neuroimaging of the cerebral fibrin accumulation in AD.

**Conclusion:**

BioClotAD neuroimaging biomarkers will allow the early detection of the pro‐thrombotic state in AD, opening a window of opportunity to delay the disease progression by personalized anticoagulant therapies.

**References**

1. Alzheimer's Association. 2021. https://www.alz.org/media/documents/alzheimers‐facts‐and‐figures.pdf.

2. Cortes‐Canteli *et al*. 2020. https://doi.org/10.1016/j.jacc.2019.10.062

3. Cortes‐Canteli, *et al*. 2015. https://doi.org/10.1016/j.neurobiolaging.2014.10.030

4. Cortes‐Canteli, *et al*. 2012. https://doi.org/10.3233/JAD‐2012‐120820

5. Cortes‐Canteli, *et al*. 2019. https://doi.org/10.1016/j.jacc.2019.07.081

6. Oliveira, Caravan. 2017. https://doi.org/10.1039/c7dt02634j

7. Sehlin, *et al*. 2019. https://doi.org/10.1007/s00259‐019‐04426‐0

